# Expression of TCR-Vβ peptides by murine bone marrow cells does not identify T-cell progenitors

**DOI:** 10.1111/jcmm.12572

**Published:** 2015-03-06

**Authors:** Janice L Abbey, Holger Karsunky, Thomas Serwold, Peter Papathanasiou, Irving L Weissman, Helen C O’Neill

**Affiliations:** a Research School of Biology, Australian National UniversityCanberra, ACT, Australia; b Institute for Stem Cell Biology and Regenerative Medicine, Stanford University School of MedicineStanford, CA, USA

**Keywords:** T-cell receptors, thymus, hematopoiesis, progenitors

## Abstract

Germline transcription has been described for both immunoglobulin and T-cell receptor (TCR) genes, raising questions of their functional significance during haematopoiesis. Previously, an immature murine T-cell line was shown to bind antibody to TCR-Vβ8.2 in absence of anti-Cβ antibody binding, and an equivalent cell subset was also identified in the mesenteric lymph node. Here, we investigate whether germline transcription and cell surface Vβ8.2 expression could therefore represent a potential marker of T-cell progenitors. Cells with the TCR phenotype of Vβ8.2^+^Cβ^−^ are found in several lymphoid sites, and among the lineage-negative (Lin^−^) fraction of hematopoietic progenitors in bone marrow (BM). Cell surface marker analysis of these cells identified subsets reflecting common lymphoid progenitors, common myeloid progenitors and multipotential progenitors. To assess whether the Lin^−^Vβ8.2^+^Cβ^−^ BM subset contains hematopoietic progenitors, cells were sorted and adoptively transferred into sub-lethally irradiated recipients. No T-cell or myeloid progeny were detected following introduction of cells *via* the intrathymic or intravenous routes. However, B-cell development was detected in spleen. This pattern of restricted *in vivo* reconstitution disputes Lin^−^Vβ8.2^+^Cβ^−^ BM cells as committed T-cell progenitors, but raises the possibility of progenitors with potential for B-cell development.

## Introduction

Germline transcription is the production of mRNA transcripts from either the unrearranged T-cell receptor (TCR) or immunoglobulin (Ig) loci that are in ‘germline’ configuration. A common view has been that production of germline transcripts is tightly coupled to rearrangement events that take place at these loci to produce a functional TCR or B-cell receptor (BCR). By this scenario, one would expect germline transcripts to be produced at VDJ recombination when the chromatin is open and fully accessible to transcriptional machinery. However, germline TCR-Vβ transcripts have been identified in early murine T cells well before the TCR-Vβ rearrangement event in RAG^−/−^ mice where no TCR rearrangement occurs 1–3, and in cell subsets and cell lines expressing fully rearranged TCR genes 2,4,5. This evidence refutes a model that associates germline transcription uniquely with the TCR rearrangement event. In addition, germline transcripts are generally thought to be sterile and non-functional. However, earlier antibody binding experiments have suggested that these transcripts encode and present a truncated TCR-Vβ molecule on the cell surface in the absence of a Cβ region. Cells of the C1-V13D line bind antibody to Vβ8.2 but not Cβ 2. A small subset of cells with the Vβ8.2^+^Cβ^−^ phenotype was later identified in the mesenteric lymph node of DBA/2J mice (∼4%). This was strain specific with a lower 1.4% found in SJL/J mice 6.

Several unusual TCR and Ig receptor structures have been identified as evidence of germline-encoded proteins and novel TCR structures. For example, germline-encoded IgV_H_ proteins have been reported 7. Dual expression of TCRαβ structures functionally responsive to *Staphylococcus* enterotoxin B have been identified in mice 8 and humans 9,10. Dual TCRγ chain receptor expression has also been reported 11, along with cell surface expression of a rearranged TCR-Vβ chain in the absence of pTα or CD3 12. TCR-Vβ expression can occur on the cell surface as a structure differing from the conventional TCRαβ receptor.

The expression of germline TCR-Vβ8 transcripts has been documented in both early B and T-cell subsets and cell lines like C1-V13D 4,6. In mice, germline-encoded TCR-Vβ is detectable in multiple lymphoid tissues including mesenteric lymph node, spleen, thymus and bone marrow (BM) 13. While *in vivo* subsets expressing Vβ8 but not Cβ determinants have been identified, there is little known about them. The developmental changes reported to occur in C1-V13D cells following intrathymic passage suggest that this cell line represents immature lymphoid cells that can differentiate along the T-cell lineage. Since germline transcripts occur during early lymphopoiesis 1,4, an important question is whether germline transcription and germline-encoded TCR proteins represent markers of T lymphoid lineage commitment. Here, we investigate the presence of Vβ8^+^Cβ^−^ cells in mouse thymus, BM, lymph node and spleen. The subset of lineage (Lin)^−^Vβ8^+^Cβ^−^ cells in BM has been further analysed for expression of markers which define hematopoietic progenitors, and their capacity to differentiate and produce T-cell progeny upon adoptive transfer in mice. While we found no evidence of T-cell reconstitution, the lymphoid characteristics of this progenitor subset were supported by specific production of mature B cells in spleen.

## Materials and methods

### Animals and tissue isolation

C57BL/Ka and C57BL/Ka-Thy1.1 (BA) mice expressing either Ly5.1 or Ly5.2 were bred and maintained in Research Animal Facility at Stanford University according to approved protocols. Male and female mice were used at 4–8 weeks of age. Mice were killed by CO_2_ asphyxiation. Spleen, thymus and BM were aseptically removed from 5 to 10 mice for preparation of cell suspensions. For isolation of hematopoietic cells from BM, femur and tibia of hind legs were removed, excess tissue discarded, and the bones crushed in a small volume of medium PBS/2%fetal calf serum. Additional medium was added until all BM cells were released away from bone fragments.

### Cell surface antibody staining

Spleen, thymus and lymph node cells were dissociated, and the cell suspension filtered through nylon mesh. Red blood cells were removed using lysis buffer (150 mM NH_4_Cl, 100 mM KHCO_3_, 0.1 mM Na_2_EDTA, pH 7.2–7.4) followed by washing in PBS/2%FCS. Cells were stained with antibody either directly with fluorochrome-conjugated antibodies, or indirectly with a purified antibody followed by a second stage conjugate. Antibodies and their specificity are shown: TCR-Vβ8.1/8.2 (MR5.2), TCR-Cβ (H57-597), Thy1.1 (19EX5), NK1.1 (PK136), B220 (RA3-6B2), Ly5.1 (ALI-4A2), Ly5.2 (A20.1.7), CD127 (A7R34), Sca-1 (E13-161-7), c-Kit (2B8), CD4 (GK1.5), CD8 (53-6.7), CD3ε (KT31.1), TCRγδ (GL3), I-A^b^ (AF6-120.1), CD11c (HL3), CD44 (IM7), CD25 (7D4), CD19 (MB19-1), Mac-1 (M1/70) and Gr-1 (8C5). All antibodies were purified from hybridoma culture supernatants with the exception of antibodies specific for CD11c, CD25, CD44, TCRγδ, I-A^b^, NK1.1, TCR-Cβ, TCR-Vβ8.1/8.2 and Ter119 purchased from BD Biosciences Pharmingen (San Jose, CA, USA). Anti-CD19 antibody was purchased from eBiosciences (San Diego, CA, USA). Secondary antibody conjugates used included Streptavidin-PE and Streptavidin-Cy7PE from Invitrogen (Carlsbad, CA, USA).

Following staining, cells were resuspended in PBS/2%FCS containing 1 μg/ml of propidium iodide (PI) to detect dead cells by flow cytometry. Normal BM cells were stained to set PI gates for sorted and depleted BM subsets. Cells were analysed for up to 5-colour staining using a FACS Vantage SE (Becton Dickinson, San Jose, CA, USA), and CellQuest Pro software (Becton Dickinson). Viable cells (PI^−^) were gated using side scatter (SSC), and investigated for marker expression or sorted for cell subset isolation. For isolation of rare cell subsets, sorted cells were checked flow cytometrically and resorted to ensure high purity.

### Lineage depletion of cells

Depletion of cells of known lineage (Lin^+^) from BM involved staining cells with purified antibodies specific for known cell lineage markers. Antibodies used were specific for Thy1.1 (19XE5), CD3ε (KT31.1), CD4 (GK1.5), CD5 (53-7.3), CD8 (53-6.7), B220 (RA3-6B2), Mac-1 (M1/70), Gr-1 (8C5) and Ter119). Following antibody staining, cells were washed and incubated with magnetic Dynabeads (#M-450) Sheep anti-Rat IgG (Dynal, Mount Waverley, VIC, Australia) at 4°C. A magnetic field was applied and the supernatant collected. The procedure was repeated. Cells were resuspended in 400 μl PBS/2%FCS containing goat F(ab’)_2_ anti-Rat IgG (H+L) conjugated to Cy5PE and incubated on ice for 20 min. Washed cells were resuspended in 400 μl of PBS/2%FCS containing 50 μl of rat IgG as a blocking antibody. Cells were incubated for 10 min on ice and the Lin^−^ cell population then stained with antibody.

### *In vivo* differentiation studies

To test whether Lin^−^Vβ8^+^Cβ^−^ cells in BM contained T-cell progenitors, sorted cells were adoptively transferred *in vivo* and their progeny developing in spleen and thymus assessed. BM cells of Lin^−^Vβ8^+^Cβ^−^ phenotype which had been sorted and resorted from BA(Ly5.1) mice were injected into C57BL/Ka-Ly5.2 sub-lethally irradiated hosts (4.75 Gy). Mice were given 500 cells either (a) intravenously through the orbital plexus, or (b) intrathymically, into a single thymic lobe 14. Control mice were given either hematopoietic stem cells (HSC; Kit^+^Lin^−^Sca^+^ KLS) or Lin^−^Vβ8^−^Cβ^−^ cells, also sorted out of BM. Post transplantation, mice were maintained on water containing polymyxin B sulphate (10^6^ U/ml) and neomycin sulphate (1.1 g/l). Mice were killed at 4 weeks, and cells stained with antibodies to detect hematopoietic cell types among donor-derived progeny. This time was chosen as a compromise between time for detection of T-cell progeny of common lymphoid progenitors (CLP) which occurs between 14 and 28 days 15, and for early development of T cells from hematopoietic stem cells or multipotential progenitors (MPP) which can be detected from as early as 3 weeks 16.

## Results

### Characterization of Vβ8^+^Cβ^*−*^ cells in lymphoid tissues

**Figure 1:**
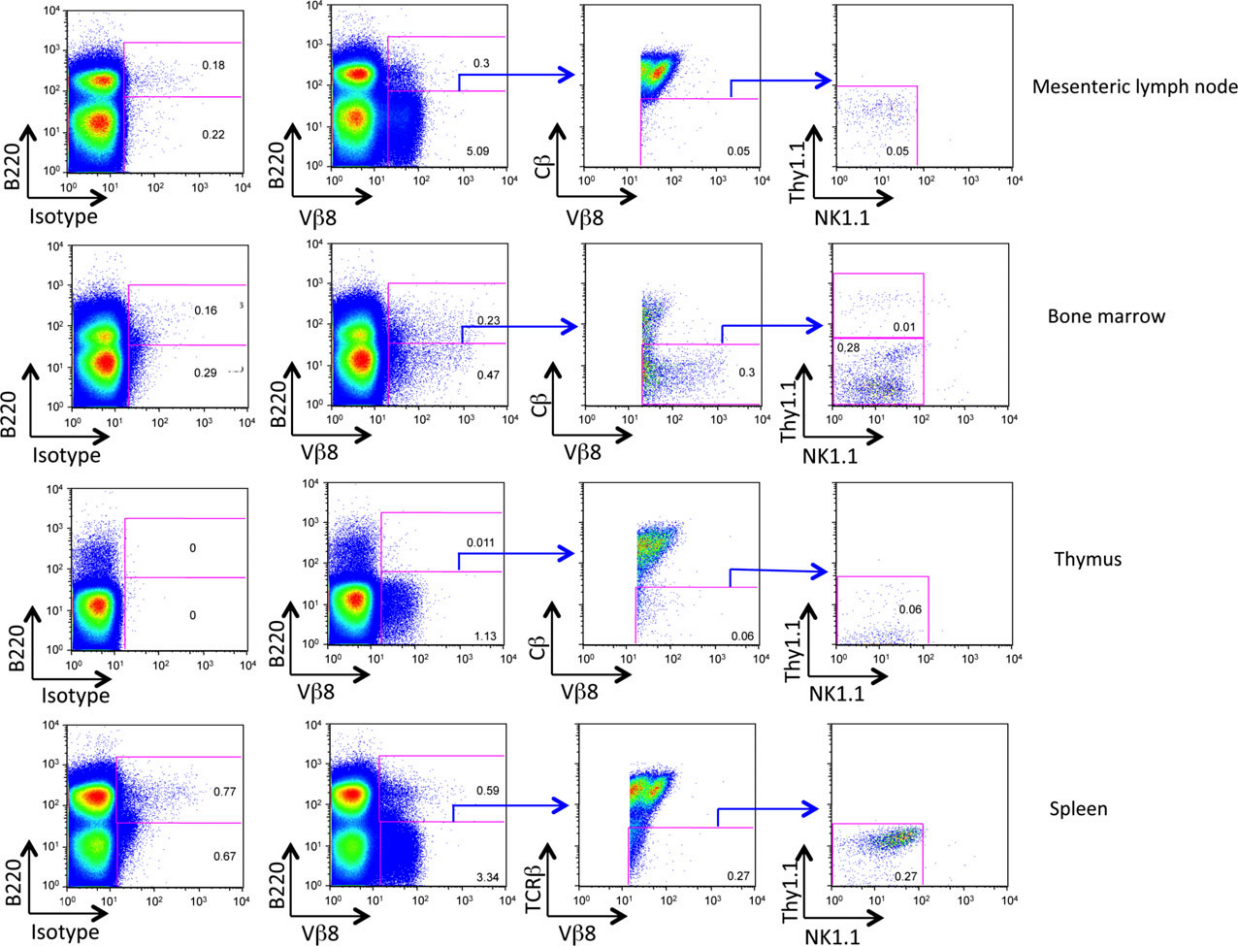
Identification of Vβ8^+^Cβ^−^ cells in murine lymphoid tissues. Cells were isolated from mesenteric lymph node, bone marrow, thymus and spleen of BA mice. Each sample was stained with fluorochrome-conjugated antibodies specific for Vβ8, Cβ, NK1.1, Thy1.2 and B220. Antibody binding was measured by flow cytometry. Live (PI^−^) B220^−^ lymphoid cells were gated prior to delineating subsets of Vβ8^+^Cβ^−^ cells also shown to be NK1.1^−^ and Thy1^−^. Numbers in quadrants reflect % positive staining cells among total viable lymphoid population in each tissue. Isotype control antibody binding was used to set gates. Representative data are shown.

The prevalence of Vβ8^+^Cβ^−^ cells was analysed flow cytometrically in thymus, BM, mesenteric lymph nodes and spleens of C57BL/6-Ly5.1 (BA) mice. To avoid any steric hindrance, cells were first incubated with anti-Cβ antibody, followed by anti-Vβ8, an isotype control antibody or no antibody. Cells were stained with antibodies specific for lineage and stem cell markers to further define the Vβ8^+^Cβ^−^ subset. Live cells were gated as PI negative (PI^−^) cells, and the Vβ8^+^Cβ^−^B220^−^ cell population identified (Fig.1). Cells showed no expression of the lineage markers NK1.1, Thy1.1 and B220, reflecting natural killer cells, T cells and B cells, respectively. In mesenteric lymph node of BA mice, 5% of cells were Vβ8^+^B220^−^. Most of these were mature Vβ8^+^Cβ^+^ T cells with 1% asVβ8^+^Cβ^−^B220^−^ cells, reflecting 0.05% of total mesenteric lymph node (Fig.1). In BM, a smaller Vβ8^+^B220^−^ population (0.5%) was identified (Fig.1), with only 60% of these cells Cβ^−^, reflecting 0.3% of total BM. It is also notable that the level of Vβ staining on Vβ8^+^Cβ^−^ cells in BM is higher than that on the small population of Vβ8^+^Cβ^+^ cells in BM. In thymus, a distinct population of 1.1% was found to be Vβ8^+^B220^−^ cells (Fig.1). Of these, only 5.5%, reflecting 0.06% of total thymus, were Cβ^−^ cells. In spleen, 3.3% of cells were Vβ8^+^B220^−^, and only 9.1% of these were Cβ^−^, reflecting 0.3% of total spleen. Across all tissues, the percentage of Cβ^−^ cells among Vβ8^+^B220^−^ cells was greatest in BM, consistent with an absence of mature T cells. One explanation is that Vβ8^+^Cβ^−^ cells originate in BM, and migrate from BM to secondary lymphoid tissues 17–21, consistent with the presence of a small number of Ly5 allotype-distinct Vβ8^+^Cβ^−^ cells in the mesenteric lymph node, thymus and spleen. We also noted the presence of a Vβ8^+^B220^+^ subset present in mesenteric lymph node (0.3%) and spleen (0.6%), not present in thymus.

### The Vβ8^+^Cβ^*−*^ subset in BM and spleen contains hematopoietic progenitors

The relationship between the Vβ8^+^Cβ^−^ subset in BM and other hematopoietic progenitor subsets was investigated flow cytometrically. Subsets in spleen were also analysed since the spleen is the source of the C1-V13D cell line, and contains hematopoietic stem/progenitor cells 22. Common lymphoid progenitors, distinguishable as c-Kit^lo^Sca-1^lo^CD127(IL-7Rα)^+^ cells 15, reflect the proximate source of T-cell progenitors in BM 23. MPP as c-Kit^hi^Sca-1^hi^CD127^−^ cells in BM also reflect a source of T-cell progenitors 24, in that they can seed BM where they develop into CLP and later produce T cells in thymus 23. On the basis of our hypothesis that Vβ8^+^Cβ^−^ cells reflect T-cell progenitors, the Vβ8^+^Cβ^−^ subsets in spleen and BM were investigated primarily for expression of markers which delineate T-cell progenitors from other hematopoietic progenitors.

**Figure 2:**
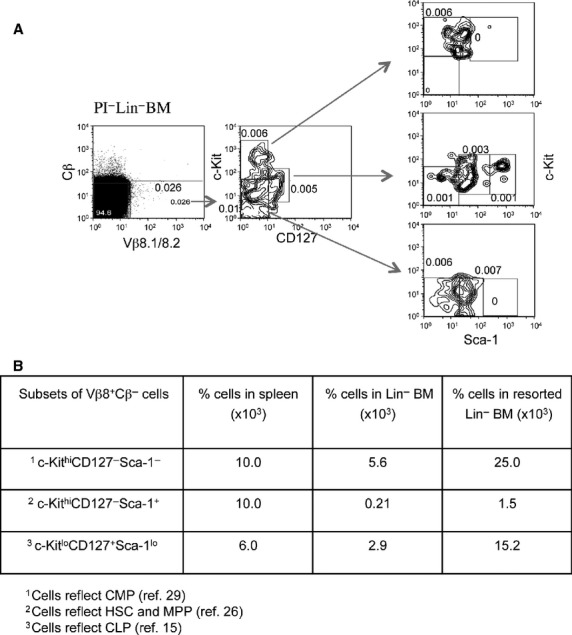
Analysis of Vβ8^+^Cβ^−^ cells among bone marrow (BM) progenitors. A population of Lin^−^ BM was prepared from BA (C57BL/Ka-Ly5.1) mice and cells were stained with fluorochrome-conjugated antibodies specific for Vβ8, Cβ, CD127, Sca-1 and c-Kit. FACS analysis was used to distinguish cell subsets. (A) Viable (PI^−^) lymphoid cells were gated initially on the basis of Side Scatter (SSC) and then Vβ8^+^Cβ^−^ cells were gated for analysis of marker expression of c-Kit, Sca-1 and CD127 markers to delineate hematopoietic progenitors. Numbers in quadrants reflect % cells staining relative to the initial gated population of viable lymphoid cells. (B) Size comparison of subsets isolated from total lineage depleted (Lin^−^) BM, resorted Lin^−^BM and spleen.

Lin^−^ BM cells were prepared and stained with antibodies to Vβ8 and Cβ to gate the Vβ8^+^Cβ^−^ subset. This population was then analysed for expression of c-Kit, Sca-1 and CD127, and distinct subsets of c-Kit^+^ hematopoietic progenitors delineated (Fig.2). Clear populations of Sca-1^lo^c-Kit^lo^CD127^+^ (CLP-like) cells, Sca-1^−^c-Kit^hi^CD127^−^ [common myeloid progenitor (CMP)-like], but almost no Sca-1^+/hi^c-Kit^+^CD127^−^ (HSC- or MPP-like) cells could be distinguished among the Vβ8^+^Cβ^−^ subset of BM. The Sca-1^+^c-Kit^−^CD127^−^ subset does not resemble progenitors and could reflect lymphoid cells. The phenotypes of CLP, CMP, MPP and HSC with respect to these markers have been described previously 15,25,26. A summary of the percentage representation of these subsets among Lin^−^ BM, resorted Lin^−^ BM and spleen is shown in Figure2. While CMP-like and CLP-like cells were present among the Vβ8^+^Cβ^−^ subset of spleen and BM, the HSC-like or MPP-like subset was less well represented among the BM subset.

This suggests that the Vβ8^+^Cβ^−^ subsets of spleen and BM could contain a range of cells reflecting hematopoietic progenitors.

### Differentiative potential of Vβ8^+^Cβ^*−*^ BM cells following adoptive transfer into mice

On the basis of these results, it was has been suggested that the Vβ8^+^Cβ^−^ subset of BM contains hematopoietic progenitors, and possibly T-cell progenitors, that develop into mature cells following adoptive transfer *in vivo*. A single time-point of 4 weeks was chosen as sufficient time post-transfer to see the rapid development of T cells from CLP, which should appear in thymus and spleen from 2 to 4 weeks. At 4 weeks, it is also possible to detect myeloid cells as the earliest progeny of any HSC and MPP developing through a CMP intermediate. Four weeks is also the earliest point at which lymphoid progeny of HSC and MPP are evident in thymus and spleen from CLP developing within BM 23.

In a first experiment, 500 resorted Lin^−^Vβ8^+^Cβ^−^ BM cells from BA (Ly5.1) mice prepared as described in Figure2 were intrathymically injected into sub-lethally irradiated (4.75 Gy) C57BL/Ka-Ly5.2 host mice. In parallel, control BM populations of sorted HSC (KLS cells: c-Kit^+^Lin^−^Sca^+^) or Lin^−^Vβ8^−^Cβ^−^ cells were prepared. Some mice were injected with PBS to provide a control for inoculation and for Ly5.1 and Ly5.2. In a second experiment, the same Lin^−^Vβ8^+^Cβ^−^ cells were given intravenously, but anti-Thy1.1 antibody was used in place of anti-CD3ε antibody for depletion of T cells, to preclude any loss of T-cell precursors on the basis of CD3ε expression. In this experiment, the Lin^−^Vβ8^−^Cβ^−^ subset containing HSC and MPP was sorted and served as a positive control for reconstitution. At 4 weeks post-transfer, mice were killed and both splenocytes and thymocytes collected and stained with antibodies specific to detect the presence of cells of different hematopoietic lineages.

**Figure 3:**
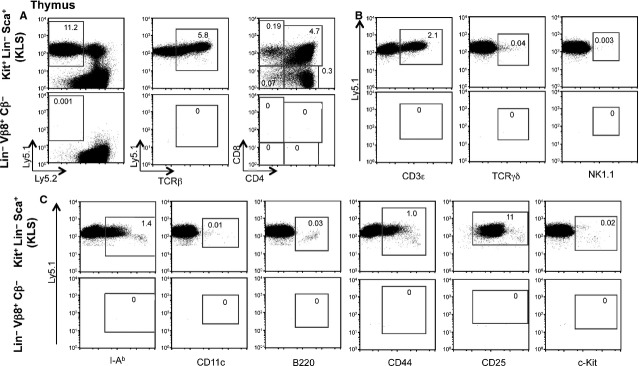
Absence of T-cell progenitors in Lin^−^Vβ8^+^Cβ^−^ bone marrow. A population of Lin^−^ BM was prepared from BA (C57BL/Ka-Ly5.1) mice. Cells were sorted twice to give c-Kit^+^Sca-1^+^Lin^−^ (KLS) and Lin^−^Vβ8^+^Cβ^−^ cells. Five hundred cells were injected intrathymically into sub-lethally irradiated (4.75 Gy) C57BL/Ka-Ly5.2 mice. After 4 weeks, thymocytes were stained in three different stainings (A–C) with anti-Ly5.1 and anti-Ly5.2 antibodies for flow cytometric detection of Ly5.1^+^ cells expressing the lineage markers TCR-Cβ, CD4, CD8, CD3ε, TCRγδ, NK1.1, I-A^b^, CD11c, B220, CD44, CD25 and c-Kit. Numbers in quadrants reflect % Ly5.1^+^ cells expressing lineage markers. The staining profile and subset analysis of a single animal are shown. Further animal analyses are summarized in Table1.

**Figure 4:**
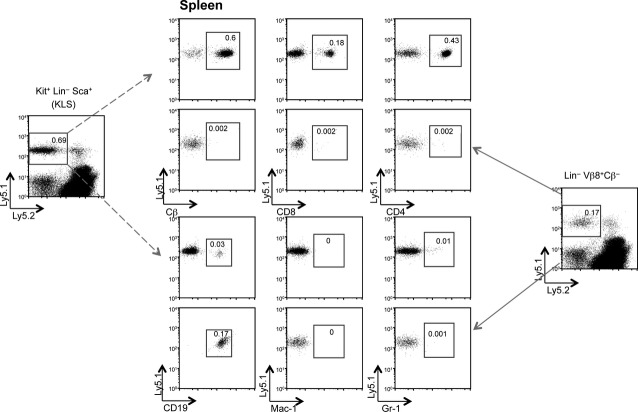
Differentiative potential of Lin^−^Vβ8^+^Cβ^−^ bone marrow cells. In the experimental protocol described in Figure3, marker expression was analysed on Ly5.1^+^ cells present among splenocytes at 4 weeks after intrathymic transfer of Lin^−^Vβ8^+^Cβ^−^ BM cells. Splenocytes were stained with antibodies for 5-colour flow cytometric detection of Ly5.1^+^ cells expressing lineage markers Cβ, CD8, CD4, CD19, Mac1 and Gr-1. Numbers in quadrants reflect % Ly5.1^+^ cells expressing lineage markers. The staining profile and subset analysis of a single animal are shown. Further animal analyses are summarized in Table1.

Adoptively transferred cells developing in spleen and thymus of the Ly5.2 hosts were first identified as viable (PI^−^) and then assessed for expression of Ly5.1 and lineage markers. Control Ly5.2 mice given PBS showed no staining for Ly5.1 (Table1). Markers used to detect cell development in thymus were specific for early progenitors (c-Kit), immature T cells (CD44 and CD25), mature αβ T cells (TCR-Cβ, CD3ε, CD4, CD8), TCR-γδ, NK and NK T cells (NK1.1), mature B cells (B220, CD19, I-A^b^) and dendritic cells (CD11c, I-A^b^). Figure3 shows the staining profile of thymocytes for one representative animal. Cell development in spleen was assessed in terms of the frequency of donor-derived T cells (Cβ, CD4, CD8), B cells (CD19), macrophages (Mac1) and granulocytes (Gr-1). Figure4 shows the staining profile of splenocytes in one representative animal.

**Table 1:** Lin^−^Vβ8^+^Cβ^−^ bone marrow progenitors lack T lineage potential

Adoptive transfer	Cells given	Spleen % Ly5.1^+^ cells	% among Ly5.1^+^ cells in spleen			Thymus % Ly5.1^+^ cells	% among Ly5.1^+^ cells in thymus	
			B cells (CD19)	T cells (Cβ)	Gran/macro (Gr-1/Mac-1)		B cells (CD19)	T cells (Cβ)
Intrathymic	Kit^+^Lin^−^Sca1^+^	0.69	4.4	87.0	1.5	11.2	5.0	51.8
Intrathymic	Lin^−^Vβ8^+^Cβ^−^	0.17	99.0	1.2	0.6	0.001	0	0
Intravenous	Lin^−^Vβ8^+^Cβ^−^	0.05	99.2	0	0	0	0	0
Intravenous*	Lin^−^Vβ8^+^Cβ^−^	0	0	0	0	0	0	0
Intrathymic	Lin^−^Vβ8^−^Cβ^−^	0.29	2.66	69.0	0.76	31.9	0	46.1
Intravenous	Lin^−^Vβ8^−^Cβ^−^	7.3	94	0.1	0.61	3.5	2.5	75†
Intravenous	Lin^−^Vβ8^−^Cβ^−^	4.7	96	0.05	0.83	3.1	0.1	89‡
Intrathymic	Nil/PBS	0	0	0	0	0	0	0
Intrasplenic	Nil/PBS	0	0	0	0	0	0	0

Bone marrow cells from BA (C57BL/Ka-Ly5.1) mice were sorted twice to give pure Kit^+^Lin^−^Sca1^+^, Lin^−^Vβ8^+^Cβ^−^ and Lin^−^Vβ8^−^Cβ^−^ subsets. 500 cells were injected intravenously or intrathymically into sub-lethally irradiated (4.75 Gy) C57BL/Ka-Ly5.2 host mice. After 4 weeks, splenocytes and thymocytes were stained with anti-Ly5.1 and anti-Ly5.2 antibodies for FACS detection of Ly5.1^+^ cells expressing the lineage markers Cβ, CD19, Mac1 and Gr-1. Thymocytes were also stained for the subset markers CD4 and CD8.

* Two of three mice given Lin^−^Vβ8^+^Cβ^−^ cells showed donor cell development.

† Thymic subsets: CD4^+^ (16.3%), CD8^+^ (4.3%), CD4^+^CD8^+^ (73%).

‡ Thymic subsets: CD4^+^ (14.0%), CD8^+^ (3.3%), CD4^+^CD8^+^ (80%).

Mice intrathymically injected with control HSC (KLS) cells showed full reconstitution of every cell lineage common to spleen or thymus as anticipated (Figs3 and 4, and Table1). Hematopoietic stem cells gave rise to predominantly CD4^+^ and CD8^+^ single-positive T cells and CD4^+^CD8^+^ double-positive T cells, and minor populations of TCR-γδ^+^ T cells, MHC-II^+^ cells which could be dendritic cells and c-Kit^+^ cells in very low number (0.02%) which could be progenitors (Fig.3). The presence of low numbers of B220^+^ B cells developing in the thymus of mice given KLS cells (0.03%) has been reported previously 25,27. Amongst mice given Lin^−^Vβ8^+^Cβ^−^ cells intrathymically, only 2 out of 3 showed reconstitution with donor cells. No T cells or B cells were detected in the thymus of donor-reconstituted mice given Lin^−^Vβ8^+^Cβ^−^ cells (Fig.3), although both showed clear B-cell development in spleen with no evidence of T cell or myeloid development in that organ (Fig.4). Since CD19 is a marker of both early B cells and more mature B cells, there results are consistent with Lin^−^Vβ8^+^Cβ^−^ cells containing a restricted B lymphoid progenitor that can develop in spleen following intravenous transfer, and can also localize in spleen following intrathymic transfer (Table1). This result contradicts our conclusion in Figure2, that the Vβ8^+^Cβ^−^ subsets in spleen could contain multipotent hematopoietic progenitors, and instead appears to contain specifically B-cell progenitors.

For mice given Lin^−^Vβ8^+^Cβ^−^ BM cells, the absence of T-cell production precludes the presence of T-cell progenitors among this subset. The absence of myeloid cell development in spleen also precludes the presence of HSC or MPP among this subset. The production of B cells and their localization in spleen but not thymus is also consistent with evidence of Vβ8^+^B220^+^ cells in peripheral mesenteric lymph node and spleen but not thymus (Fig.1). Since Lin^−^Vβ8^+^Cβ^−^ cells develop into B cells in spleen following either intravenous or intrathymic inoculation, it is not yet clear whether splenic B cells derive from progenitors that differentiate within the thymus and then enter spleen, or from progenitors which differentiate within spleen itself. Overall, our reconstitution experiments indicate the presence of a B cell restricted lymphoid progenitor among the Lin^−^Vβ8^+^Cβ^−^ subset of BM. The inability to generate T-cell progeny following intrathymic inoculation of progenitors also supports the argument that this subset contains B-cell progenitors and not T-cell progenitors. The absence of myeloid progeny also demonstrates an absence of HSC or MPP among the Lin^−^Vβ8^+^Cβ^−^ subset of BM.

## Discussion

Here, the hypothesis is investigated that Vβ8^+^Cβ^−^ cells in BM represent T-cell progenitors and that the expressed Vβ8 peptide encoded from germline genes is a marker of these cells. Vβ8^+^Cβ^−^ non-B cells (B220^−^) were identified in several organs including thymus, mesenteric lymph nodes, BM and spleen of mice. To some extent these subsets resemble C1-V13D, described previously as an immature spleen-derived T-cell line expressing TCR-Vβ8.2 peptides as well as germline Vβ8.2 transcripts 28. The Vβ8^+^Cβ^−^ subset of BM and spleen was therefore analysed for expression of c-Kit, Sca-1 and CD127 markers, which distinguish CLP from other progenitor populations in BM. An initial study of c-Kit, CD127 and Sca-1 expression on a gated Vβ8^+^Cβ^−^ subset of whole spleen and BM revealed multiple sub-populations phenotypically resembling HSC, MPP, CMP and CLP.

The hypothesis that the Vβ8^+^Cβ^−^ subset contains lymphoid progenitors was based on transcriptional evidence that both early T and B cells express *GL-Vβ8.2* transcripts 4,6. Following both intrathymic and intravenous transfer of Lin^−^Vβ8^+^Cβ^−^ cells isolated from BM, no development of T cells was detected in thymus or spleen, and further development of Lin^−^Vβ8^+^Cβ^−^ BM cells was found to be limited to B lymphopoiesis. While B cells can arise in low numbers as an endogenous thymic cell population 27,29, Vβ8^+^Cβ^−^ B cells were instead detected in spleen in the absence of any thymic B-cell development. It is not yet possible to determine whether development of these B cells occurred in spleen itself, or was dependent on lodgement of progenitors in BM.

Results presented here contrast with previous evidence that the C1-V13D cell line, representing a splenic Vβ8^+^Cβ^−^ cell line, can undergo some T-cell differentiation upon intrathymic transfer showing TCRαβ expression 28. It is possible that the Lin^−^Vβ8^+^Cβ^−^ subset from BM contains lymphoid progenitors with capacity to differentiate into only B cells, while a similar or equivalent subset resident in spleen may have T-cell differentiative potential. Such a model invokes a heterogeneous population of progenitors in mice expressing germline Vβ8 peptide. This is supported by evidence for expression of *GL-Vβ8.2* transcripts in many cell types including early T cells, early B cells, BM progenitors and early dendritic cells (Abbey & O’Neill, unpublished data). Indeed, C1-V13D has been shown to express low levels of early T-cell markers including CD3ε 28. It is also important to note that lineage depletion using anti-CD3ε antibody would also deplete CLP known to express this marker 15. However, depletion with anti-Thy1.1 did not reveal a CLP with T-cell differentiative capacity even after intrathymic inoculation. The Lin^−^Vβ8^+^Cβ^−^ BM subset therefore appears to contain a later lymphoid progenitor committed to the B-cell lineage.

The Lin^−^Vβ8^+^Cβ^−^ BM population does not appear to contain bipotent CLP-like cells with capacity to differentiate into both T and B cells. In terms of bipotent lymphoid potential, thymic DN1 cells are known to be heterogeneous with specific sub-populations retaining ability to give rise to B cells 30. While bipotent T and B progenitors can give rise to B cells in thymus, this does not appear to be the case for the Lin^−^Vβ8^+^Cβ^−^ BM subset 27,29. In fact, the progenitor described here appears to have properties more closely resembling the CLP-2 cell type, which can seed thymus and differentiate into B cells 31. However since the Lin^−^Vβ8^+^Cβ^−^ cell subset does not express B220, it is distinct from the CLP-2 population identified in BM as a B220^+^ cell 31.

Mesenteric lymph nodes may also be an important source of Vβ8^+^Cβ^−^ cells 12. Mesenteric lymph nodes have also been described as a site for extra-thymic T-cell development 17–21. Consistent with this evidence is the finding that subsets of Vβ8^+^Cβ^−^B220^+^ cells exist in BM (0.07%) and mesenteric lymph node (0.12%) of BA mice, but not in spleen and thymus (Fig.1). A further question to be addressed is whether a Vβ8^+^Cβ^−^B220^−^ subset is a precursor of Vβ8^+^Cβ^−^B220^+^ cells in these tissues. The relationship between Vβ8^+^Cβ^−^B220^+^ subsets detectable in these tissues and the B-cell populations produced in reconstitution studies also needs to be addressed further.

Evidence for germline transcription of *TCR-Vβ* genes leading to expression of a cell surface peptide in B-cell progenitors raises questions of their functional role. Models for function should take into account the prevalence of Vβ8 peptide-expressing cells in multiple organs, and in both mature and immature lymphoid cells. Many attempts to investigate cell surface expression of germline TCR-Vβ8.2 expression on the surface of TCR-Cβ^−^ cells by immunoprecipitation and Western blotting have been unsuccessful. Preliminary experiments have shown that this protein is not GPI-anchored on the cell surface (Abbey & O’Neill, unpublished data). It is possible that a truncated TCR-β chain with no transmembrane region is expressed only and then is secreted by cells. Such transitional expression could also account for the variable expression reported for TCR-Vβ8.2 on the surface of C1-V13D cells 2. Transfection of germline *TCR-Vβ8.2* genes into C1-V13D cells was used to overcome this problem in demonstrating cell surface expression 32. Candidate molecules that could be involved in a cell surface complex with TCR-Vβ8.2 could include pTα and CD3ε, each of which is expressed by C1-V13D (Abbey & O’Neill, unpublished data).

One possible role for germline-encoded TCR-Vβ peptides could be generation of tolerance to T cells *in vivo*. A substantial level of diversity exists within the TCR and BCR repertoire against which developing T and B lymphocytes must be tolerized. Therefore, germline-encoded Ig or TCR proteins may play an antigenic role in this process within thymus for T-cell development, or within BM, spleen and lymph node for B-cell development. The expression of multiple germline *TCR-Vβ* transcripts in the cloned C1-V13D cell line also supports this model 5.
